# Supplementary material for: Modeling glioblastoma heterogeneity as a dynamic network of cell states
Source: Mol Syst Biol. 2021 Sep 16;17(9):e10105. doi: 10.15252/msb.202010105 (PMC8444284; doi:10.15252/msb.202010105)
Supplement: Supplementary file 6 — Source Data for Figure 5 [file MSB-17-e10105-s004.zip › Figure5A_sourcedata/GSEA_3017/nosigdb_stateE.GseaPreranked.1622795031093/NB_KI_CLUSTER17.html]

Details for gene set NB\_KI\_CLUSTER17[GSEA]

|  || Dataset | state63017 |
| Phenotype | NoPhenotypeAvailable |
| Upregulated in class | na\_pos |
| GeneSet | NB\_KI\_CLUSTER17 |
| Enrichment Score (ES) | 0.6760104 |
| Normalized Enrichment Score (NES) | 3.5246756 |
| Nominal p-value | 0.0 |
| FDR q-value | 0.0 |
| FWER p-Value | 0.0 |
Table: GSEA Results Summary

  

Fig 1: Enrichment plot: NB\_KI\_CLUSTER17      
 Profile of the Running ES Score & Positions of GeneSet Members on the Rank Ordered List

  

| PROBE | GENE SYMBOL | GENE\_TITLE | RANK IN GENE LIST | RANK METRIC SCORE | RUNNING ES | CORE ENRICHMENT || 1 | PMP22 |  |  | 0 | 1.393 | 0.1248 | Yes |
| 2 | RCAN1 |  |  | 17 | 0.750 | 0.1746 | Yes |
| 3 | ANXA2 |  |  | 18 | 0.733 | 0.2402 | Yes |
| 4 | CNN3 |  |  | 24 | 0.702 | 0.2976 | Yes |
| 5 | EMP1 |  |  | 40 | 0.584 | 0.3336 | Yes |
| 6 | PMEPA1 |  |  | 44 | 0.544 | 0.3791 | Yes |
| 7 | CD59 |  |  | 89 | 0.418 | 0.3687 | Yes |
| 8 | GPM6B |  |  | 105 | 0.391 | 0.3875 | Yes |
| 9 | FNDC3B |  |  | 123 | 0.354 | 0.4007 | Yes |
| 10 | AHNAK |  |  | 125 | 0.351 | 0.4311 | Yes |
| 11 | VMP1 |  |  | 127 | 0.351 | 0.4614 | Yes |
| 12 | PLPP3 |  |  | 139 | 0.342 | 0.4801 | Yes |
| 13 | RHOB |  |  | 148 | 0.335 | 0.5014 | Yes |
| 14 | PDLIM2 |  |  | 155 | 0.329 | 0.5244 | Yes |
| 15 | SPATS2L |  |  | 163 | 0.323 | 0.5457 | Yes |
| 16 | JUN |  |  | 172 | 0.316 | 0.5654 | Yes |
| 17 | MIR99AHG |  |  | 193 | 0.300 | 0.5706 | Yes |
| 18 | SPTBN1 |  |  | 194 | 0.300 | 0.5974 | Yes |
| 19 | FTH1 |  |  | 204 | 0.293 | 0.6139 | Yes |
| 20 | GNG11 |  |  | 210 | 0.289 | 0.6343 | Yes |
| 21 | DAG1 |  |  | 228 | 0.279 | 0.6409 | Yes |
| 22 | QKI |  |  | 241 | 0.267 | 0.6518 | Yes |
| 23 | TNFRSF12A |  |  | 262 | 0.257 | 0.6530 | Yes |
| 24 | WWTR1 |  |  | 263 | 0.256 | 0.6760 | Yes |
| 25 | EPB41L2 |  |  | 488 | -0.310 | 0.4605 | No |
| 26 | EGR1 |  |  | 656 | -0.398 | 0.3149 | No |
Table: GSEA details [plain text format]

  

Fig 2: NB\_KI\_CLUSTER17: Random ES distribution      
 Gene set null distribution of ES for **NB\_KI\_CLUSTER17**
